# HMST-Seq-Analyzer: A new python tool for differential methylation and hydroxymethylation analysis in various DNA methylation sequencing data

**DOI:** 10.1016/j.csbj.2020.09.038

**Published:** 2020-10-10

**Authors:** Amna Farooq, Sindre Grønmyr, Omer Ali, Torbjørn Rognes, Katja Scheffler, Magnar Bjørås, Junbai Wang

**Affiliations:** a Department of Pathology, Oslo University Hospital – Norwegian Radium Hospital, Oslo, Norway; b Department of Informatics, University of Oslo, Oslo, Norway; c Institute for Clinical and Molecular Medicine, Norwegian University of Science and Technology, Trondheim, Norway; d Department of Microbiology, Oslo University Hospital, Oslo, Norway; e Department of Microbiology, University of Oslo, Oslo, Norway; f Department of Neuromedicine and Movement Science and Department of Clinical and Molecular Medicine, Norwegian University of Science and Technology, Trondheim, Norway; g Department of Neurology and Department of Laboratory Medicine, St. Olavs Hospital, Trondheim, Norway

**Keywords:** Methylation analysis, Hydroxy methylation, Differential methylation, Hydroxymethylation-and methylation-sensitive tag sequencing, Whole genome bisulfite sequencing

## Abstract

DNA methylation (5mC) and hydroxymethylation (5hmC) are chemical modifications of cytosine bases which play a crucial role in epigenetic gene regulation. However, cost, data complexity and unavailability of comprehensive analytical tools is one of the major challenges in exploring these epigenetic marks. Hydroxymethylation-and Methylation-Sensitive Tag sequencing (HMST-seq) is one of the most cost-effective techniques that enables simultaneous detection of 5mC and 5hmC at single base pair resolution. We present HMST-Seq-Analyzer as a comprehensive and robust method for performing simultaneous differential methylation analysis on 5mC and 5hmC data sets. HMST-Seq-Analyzer can detect Differentially Methylated Regions (DMRs), annotate them, give a visual overview of methylation status and also perform preliminary quality check on the data. In addition to HMST-Seq, our tool can be used on whole-genome bisulfite sequencing (WGBS) and reduced representation bisulfite sequencing (RRBS) data sets as well. The tool is written in Python with capacity to process data in parallel and is available at (https://hmst-seq.github.io/hmst/).

## Graphical abstract

**Figure:**
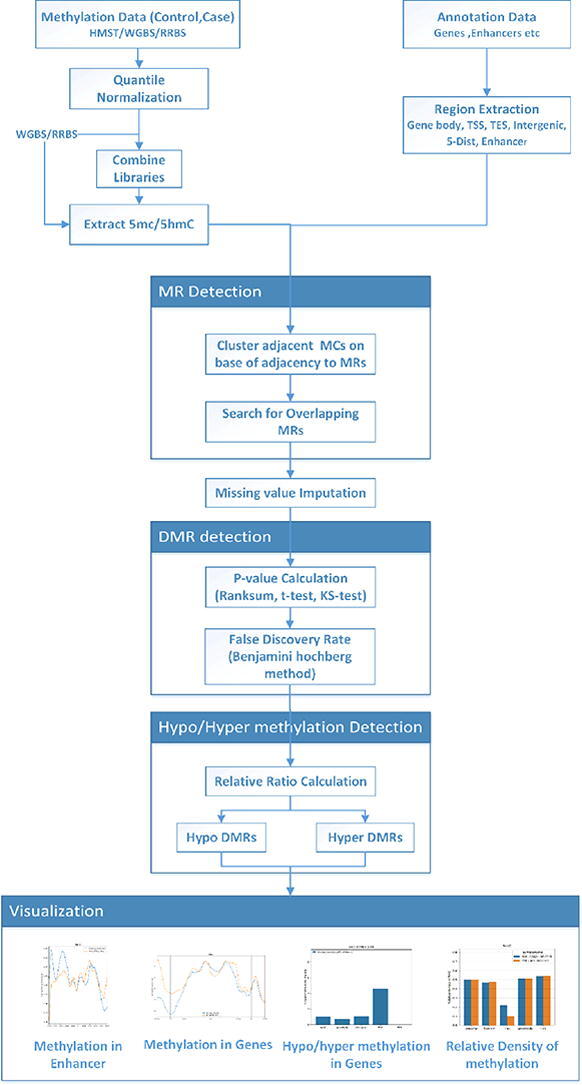

## 1 Introduction

Epigenetic DNA methylation provides an additional layer for controlling cellular processes. It is the most stable epigenetic mark that plays a significant role in gene regulation with impact on health and disease [1]. Nevertheless, DNA methylation varies in response to cell differentiation, disease, and environmental factors. In vertebrates, 5-methylcytosine (5mC) is the most abundant epigenetic mark. It is usually found in CpG context, where 5mC can be iteratively oxidized by TET proteins to 5-hydroxymethylcytocine (5hmC), the second most abundant epigenetic mark in vertebrates [2]. The aforementioned two methylation marks have essential roles in the development and regulation of cellular processes [3]. Especially, abnormal methylation patterns have been observed in many human diseases and can be used in clinical outcome predictions [4]. Hence, correct profiling of DNA methylation in a genome is key to understand the contribution of epigenetics in gene regulation.

Nowadays, bisulfite treatment is considered as the most effective way of targeting DNA methylation [5]. Whole-genome bisulfite sequencing (WGBS) is the most comprehensive protocol for measuring genome-wide single-base-pair resolution methylation, hence making it the golden standard technique [6] for studying genomic DNA methylation. Some studies in vertebrate genomes (e.g., birds and fishes) recommend 15X of sequencing depth for WGBS experiments [7], [8]. Others such as the NIH Roadmap Epigenomics Projects advise coverage of 30X (combined coverage of 2 replicates). Similarly, the ENCODE project and the International Human Epigenome Consortium (IHEC) recommend users to submit experimental data with sequencing depth of 30X for WGBS [9], [10]. To achieve 30X sequencing depth of human sized genome, ~180 GB of sequencing data is produced in WGBS experiments [11]. The data size will be reduced in case of organisms with a smaller genome size [8]. Alternatively, an enhanced protocol (HiSeq X Ten) offers improved cost effectiveness and coverage [12] for WGBS experiments. However, to achieve the claimed low cost, all the HiSeq X Ten machines are required to be run on full capacity. In addition to this fact, the total price of the system makes it suitable for larger institutes only [13] Nevertheless, WGBS remains an expensive experiment with huge data handling and processing requirements for downstream analysis. This highlights the need of enrichment-based techniques, which offer a fair trade between coverage and cost.

Reduced representation bisulfite sequencing (RRBS) is a cost-effective enrichment-based sequencing method to target DNA methylation. RRBS reduces sequencing requirements by targeting CpG rich genomic regions only [14]. RRBS produces much less data and reduces the experimental cost significantly. Unfortunately, both WGBS and RRBS are unable to distinguish between 5mC and 5hmC on DNA. Therefore, hydroxymethylation and methylation-sensitive tag sequencing (HMST-seq) was proposed to detect both 5mC and 5hmC on DNA sequences simultaneously [11]. HMST-seq takes advantage of sequence specific DNA restriction endonucleases: for example, HpaII can cleave unmodified cytosine only while MspI cleaves at both 5mC and 5hmC. Moreover, β-glucosyltransferase (β-GT) can transfer glucose to 5hmC which will block MspI digestion. By combining these enzymatic reactions, HMST-seq generates three tag libraries, which can be used to determine methylation and hydroxymethylation abundance in a sample. The first library contains information of unmodified, methylated and hydroxymethylated cytosines, generated by MspI digestion only. The second library refers to unmodified and methylated cytosines generated by glucosylation of 5hmC and subsequent MspI digestion. The third library contains only unmodified cytosines generated by HpaII digestion. HMST-seq not only targets both 5mC and 5hmC in Msp1 sites (5′-CCGG-3′) at single base resolution but also generates only ~5 GB data to achieve a 30X sequencing depth. Since HMST-seq relies on specific restriction enzymes, it is limited to regions with CCGG sites, thus covering approximately 4–7% CpG dinucleotides distributed throughout the vertebrate genome. However, it has recently been demonstrated that locations of CCGGs largely reflects those of all CpGs in the genome [15] and that epigenetic profiling by using methylation- and hydroxymethylation-sensitive restriction enzymes can successfully address fundamental biological questions [16], [17], [18], [19]. In contrast to WGBS sequencing, HMST-seq is distinguishing between 5mC and 5hmC providing additional information about the dynamics of oxidative demethylation. The cost, coverage and detection of both 5mC and 5hmC on DNA sequences make HMST-seq an attractive tool for biologists to perform whole-genome methylation studies with large sample sizes.

With the advancement of high throughput sequencing technologies, data generation rate has outpaced the Moore’s law [20] while data analysis still remains a challenge. There are, however, several tools available for differential methylation analysis (e.g., MethylKit, MethylSig, and BSmooth [21], [22], [23]). While the majority of tools are tailored towards 5mC only a few of them focus on 5hmC. Especially, none of the publicly available tools for differential methylation analysis focuses on both 5mC and 5hmC that are generated by the HMST-seq. Though the methylation data analysis pipeline MINT integrates several tools to process both methylation and hydroxymethylation data, it cannot be applied on HMST-seq library tag data [24]. As mentioned earlier, HMST-seq outputs tag counts for three libraries separately and requires processing to methylation and hydroxymethylation levels after wards, this facility is not available in previous tools like MethylKit, BSmooth or MINT pipleine. Thus, we have developed a new differential methylation analysis pipeline called HMST-Seq-Analyzer, which is a user-friendly command line Python package. Though the differential methylation analysis is similar between 5mC and 5hmC at the genome-wide scale, the pre-processing of HMST-Seq data for 5mC and 5hmC is quite different. For example, the methylation and hydroxymethylation levels are calculated by taking ratio of tag counts from three libraries. Such difference in the low-level methylation analysis is taken care by HMST-Seq-Analyzer automatically. Especially, the methylation analysis of both 5mC and 5hmC is done in a single run in the package, which simplifies the illustration and interpretation of results. The package is optimized to process huge data sets from either HMST-seq or WGBS by using parallel computation, as well as data from RRBS. HMST-Seq-Analyzer implements a methylated region (MR) search method similar to one that has been previously published in [11] to detect differentially MRs (DMRs) from HMST-seq data. Two slightly modified search methods to define MRs are available to suite the nature of different data sets. For instance, for larger data sets coming from WGBS experiments, pipeline allows tiling window analysis to efficiently deal will large number of methylated sites. Multiple statistics test are also available to search DMRs to accommodate differences in nature of data generated by different methylation detection platforms. All detected DMRs are automatically annotated to the reference genome based on the refFlat file from UCSC Genome Browser [25]. Moreover, the package provides a simple statistical summary of the distribution of methylation in various genomic regions (e.g., transcription start site (TSS), transcription end site (TES), gene body, intergenic, 5′ distance region, and other regions like enhancers). Finally, it also provides lists of hyper- and hypo-DMRs annotated to different genomic regions.

## 2 Material and methods

**Fig. 1:**
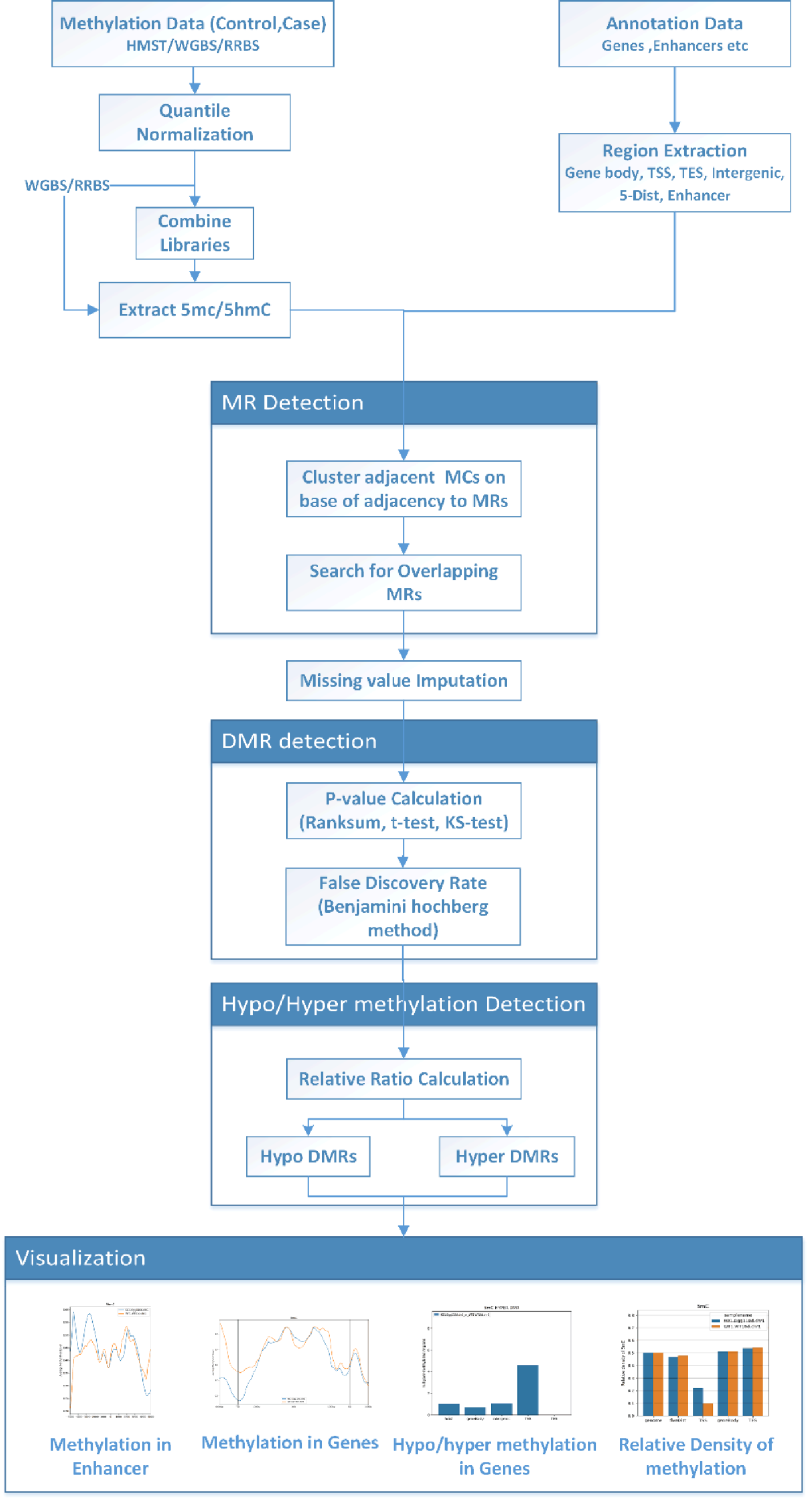
Detailed scheme of flow of HMST-Seq-Analyzer pipeline. HMST-Seq-Analyzer detects differential methylation in three major phases: 1) preprocessing of data where it performs annotation and extracts methylated regions from predefined regions (e.g., TSS, TES, enhancer, gene body, 5′ distance region and intergenic regions); 2) searches for MRs with same locus in the case and control samples, finds DMRs (e.g. using Wilcoxon Ranksum, Kolmogorov–Smirnov test or T-test), and categorizes them into Hyper/Hypo-methylated DMRs; 3) visualizes graphical summary of analysis results, exports the results in form of list of Hyper/Hypo DMRs and gene annotation.

HMST-Seq-Analyzer is a new Python package, that employs robust statistical methods for differential methylation analysis in whole-genome DNA sequencing data such as HMST-Seq, WGBS, or RRBS. Although the pipeline is optimized for HMST-seq data, it can be easily applied on either WGBS or RRBS data by simply adjusting the default MR search parameters and the DMR search method. HMST-Seq-Analyzer conducts differential methylation analysis using three steps. Generally, differential methylation detection can be done at multiple genomic resolutions like non-CpG (CHG/CHH), CpG, genome wide tiles or at annotated regions. However, in case of single base pair resolution data coming from techniques like HMST, WGBS and RRBS, differential methylation detection at base pair level becomes a computationally exhaustive task. For two reasons, the best approach is to perform annotated genome analysis; first, use of external genome annotation data focuses the analysis on those methylated regions which can possibly act as epigenetic regulatory switches and second, it reduces the search space, which is otherwise extremely dense. Hence, HMST-Seq-Analyzer tries to minimize the computational burden, by first extracting methylation regions (MR) from predefined genomic areas (e.g., TSS, TES, enhancer, gene body, 5′ distance region and intergenic regions) that may act as epigenetic regulatory switches. In the second step, it filters MRs in parallel, which further narrows down the total search space and computational cost. Finally, differentially methylated regions (DMRs) in predefined genomic areas are predicted by robust statistical tests (e.g., Wilcoxon rank-sum test) between the control and the reference samples. Fig. 1 summarizes the workflow of HMST-Seq-Analyzer. It is divided into eight discrete modules, where the default settings for the parameters are aimed for HMST-seq data but parameters are flexible to accommodate RRBS and WGBS data in the package. A more detailed description of HMST-Seq-Analyzer can be found in the following subsections.

### 2.1 Quality control

Before running the pipeline, users can check input data quality by calculating the average of read count distribution among the samples. It is preferable to have majority of the reads distribution above 15X coverage in a sample to ensure reliable prediction of DMR [7] as recommended by the ENCODE project [9]. This facility is provided as an additional script in the toolbox in the demo data set. Apart from the average read count, the toolbox also offers preliminary cleaning and processing of input files.

### 2.2 Pre-processing

By default setting HMST-Seq-Analyzer extracts five types of regions from a given reference genome refFlat file: TSS, gene body, TES, intergenic, and 5′ distance region of each gene. A detailed illustration of these five regions is shown in SFig. 1, where Gene body is defined between TSS and TES. TSS and TES are ± 1 kb (default) from the original TSS and TES, respectively. The 5′distance is from 10 kb to 100 kb upstream (default) of the TSS. The intergenic regions consider the whole genome except for TSS, TES and gene body. In this way, the pipeline includes whole genome in differential methylation analysis. The lengths of TSS, TES, 5′distance and intergenic regions can be adjusted by the user in the pipeline. An additional enhancer option can also be used by any other regions of choice, provided that chromosomal coordinates are given in simple bed format. The gene annotation task is required only once if the same genomic regions will be used across multiple experiments. Predicting DMRs from aforementioned five types of genomic regions may help us to identify putative functional DMRs in downstream analysis. For HMST-Seq data, if input tag counts are not normalized across sample, then a quantile normalization method can be applied. Subsequently, an abundance of 5mC/5hmC level from HMST-seq can be determined by the ratio between the normalized tag counts of two libraries (e.g, between BGT (β–glucosyltransferase – to detect 5hmC) and HpaII for 5mC; between MspI and BGT for 5hmC). For RRBS and WGBS, a direct input of methylation levels and the corresponding chromosome positions from a bed formatted file is requested. The pipeline offers to switch between the two major types of data sets: methylation level based, i.e. WGBS/RRBS, and library tag count based, i.e. HMST-Seq.

### 2.3 Differential methylation analysis

In HMST-Seq-Analyzer, there are two major steps to identify DMRs from genome-wide DNA methylation sequencing data: 1) Searching for candidate methylation regions (MR); 2) Identifying significantly differentially methylated regions (DMRs) with robust statistical tests.

#### 2.3.1 Searching for methylated regions

Methylated and hydroxymethylated regions can vary in size from a single methylated base pair to an entire gene locus, depending on the biological question of interest. Although single methylated CpG is reported to affect gene expression regulation [26] and disease risk [27], the majority of DMRs are reported to range from a few hundred bases to a few kilobases which may be biologically more interesting [28]. Especially, DMRs are known to regulate cell-type specific transcriptional repression of genes [29], and this size range complements with the size of other gene regulatory regions such as enhancers. Though HMST-seq data are limited to HpaII restriction sites, it has recently been shown that locations of CCGGs are evenly distributed comparable to those of all CpGs in the genome, and methylation level determined in specific genomic regions (e.g. promoter) highly correlate with gene expression [15]. Thus, searching for methylated regions (MR) makes sense for HMST-seq data. Moreover, epigenetic profiling by using methylation- and hydroxymethylation-sensitive restriction enzymes successfully addresses fundamental biological questions [17], [18], [19].

In HMST-Seq-Analyzer, an MR is defined as a cluster of 5mC or 5hmC sites at a locus. The search for MRs is only carried out in certain defined genomic regions (e.g., gene body, TSS, or enhancer etc). There are two major stringency parameters in the MR search method: the number of consecutive sites and the distance of adjacent methylated sites. For HMST-Seq data, this is similar to the previous publication [11], where any clusters of methylated sites would be classified as MR: (1) if there are at least *N* (the default is 5 for gene body/intergenic/5′Distance and 3 for TSS/TES) 5mC/5hmC sites in the region, and (2) the distance between two consecutive methylated sites is not greater than *a* bp (the default is 2000 bp). CpGs in an MR can have increasing or decreasing trend of methylation and this trend can be similar or mixed in the case and control samples. HMST-Seq-Analyzer allows restricting the DMR search to same methylation trend by using an additional parameter in the DMR search function (−isST = 1 or 0 will force all CpG sites in the same MR shall have the same or mixed changing trend, respectively) [11]. These parameters can be easily changed by command line options. For WGBS and RRBS data, slightly modified parameters can be used to search MRs (e.g., N = 3 and a = 2000 bp for WGBS; N = 2 and a = 200 bp for RRBS). Alternatively, the package also provides an equal sized sliding window bin approach which can be applied on the data to search for MRs. This option is suggested for large and dense data set such as WGBS.

#### 2.3.2 Identification of differentially methylated regions

The current package performs pair-wise or multiple samples comparison for differential methylation analysis (e.g., one control versus one case, or one/multiple controls versus one/multiple cases). Therefore, MRs or methylation sites need to be available in both conditions before performing a significant differential methylation analysis. Unfortunately, due to DNA sequencing variation and many other uncontrollable reasons in methylation experiments, some methylation signals may be missing in one of the conditions, even if from technical replicates. This is a missing value problem in sequencing data analysis, which is the first challenge in differential methylation analysis. A missing value indicates that methylation level at a position is not recorded as an experimental error or it was an unmethylated state and a methylation level was recorded as zero. There are three possible ways to deal with the missing value: ignoring, deletion, or imputation. The first two options cannot be good options because they can result in loss of an important DMR. Therefore, all missing values are imputed in the current pipeline before identifying DMRs. For less computational cost, the missing values can simply be replaced by the median of methylations in an MR or by zeros (default, because it resembles a possible real unmethylated state). Alternatively, a nearest neighbour data imputation method can be applied on an MR with missing values, but this method will have heavy computational burden. After finding MRs sharing the same locus in both conditions and imputing the missing values, the pipeline applies the Wilcoxon Rank-sum test to evaluate the significance of differentially methylated MRs as used by many similar studies [11], [18], [30]. There are three versions of the Wilcoxon Rank-sum test available in the pipeline: Pranksum, Mranksum, and Rranksum. Pranksum is a Python implementation of the *P-value* computation for the Wilcoxon rank-sum test:

$$p=\left\{\begin{array}{c} exact\;\;\;\;\;\;\;\;\;\;\;\;\;\;\;\;\;\;\;\;\;\;\;\;\;if\;n_{x}<10\\ mannwhitneyu\;\;\;\;\;\;\;\;\;otherwise \end{array}\right)$$

where $n_{x}$ is the number of 5mC/5hmC sites in an MR, exact is a Python implementation of the exact enumeration of P-value [31] when the sample size is small (this is a contribution of this research work in the pipeline), and mannwhitneyu is a function from Python library scipy.stats that performs a two-sided Mann-Whitney *U* test or Wilcoxon rank-sum test. The exact calculation of P-value is recommended when there are less than 10 methylation sites in a MR. For a large sample size, an approximation of P-value can be obtained from Python’s Mann-Whitney *U* test function. Mranksum is a Matlab version of rank-sum test, which is computed as following:

$$p=\left\{\begin{array}{c} \mathrm{exact}\;\;\;\;\;\;\;\;\;\;\;\;if\;\min\left(n_{x},n_{y}\right)<10\;and\;n_{x}+n_{y}<20\\ approximate\;\;\;\;\;\;\;\;\;\;\;\;\;\;\;\;\;\;\;\;\;\;\;\;\;\;\;\;\;\;\;\;\;\;\;\;\;\;\;\;\;\;\;\;otherwise \end{array}\right)$$

where exact is the exact computation of the P-value when sample size is small, and *approximate* is the Matlab function of the two-sided Wilcoxon rank-sum test. Hence, the pipeline also offers Rranksum, which is the wilcox.test function of R. It computes P-values as follows:

$$p=\left\{\begin{array}{c} \mathrm{exact}\;\;\;\;\;\;\;\;\;\;\;\;\;\;if\;n_{x}<50\;and\;n_{y}<20\;and\;no\;ties\\ approximate\;\;\;\;\;\;\;\;\;\;\;\;\;\;\;\;\;\;\;\;\;\;\;\;\;\;\;\;\;\;\;\;\;\;\;\;\;\;\;\;\;\;\;otherwise \end{array}\right)$$

However, the R version of the rank-sum test does not have tie correction and is much slower than both Matlab and Python implementation. MATLAB is a commercial license software that users might not have access to. A performance comparison of the three methods for DMR detection is provided in SFigs. 2 and 3. One of these three methods should be chosen based on the resources available. Alternative statistic test methods are also implemented in the HMST-Seq-Analyzer for identifying DMRs such as two sampled T-test [22] and Kolmogorov-Smirnov test [32]. In the future, more advanced methods (e.g. the Beta-binomial method [23]) will be considered in the pipeline. Pranksum is the default setting in HMST-Seq-Analyzer.

For each MR, the significance of differential methylation between the two conditions is evaluated by a *P-value*. Correction of the *P-value* by either without (default) or with Benjamini-Hochberg false discovery rate [33] is implemented in the package. MRs with P-values crossing the predefined threshold (e.g., p < 0.05 by default) are considered as DMRs. Here, an identified DMR can either exhibit an increase or a decrease in methylation levels between the two conditions, termed as hyper or hypomethylation, respectively. For that reason, a relative ratio (*rratio*) approach [34] is used to distinguish between the Hyper-DMRs and the Hypo-DMRs. It is given by the following formula:

$$\mathrm{rratio}=\frac{\mu_{\mathrm{KO}}-\mu_{\mathrm{WT}}}{\left(\frac{\mu_{\mathrm{KO}}+\mu_{\mathrm{WT}}}{2}\right)}$$

where µ_KO_ is the median of the original methylated levels in the case (KO) data, and µ_WT_ is the median of the original methylated levels in the control (WT) data. A DMR is considered as hypermethylated or hypomethylated when the *rratio* is greater than or smaller than zero, respectively. As an option, users can also plot all DMRs to investigate their quality or may run the DMR search function again by using new parameters (e.g., a new P-value threshold or a new P-value correction method).

### 2.4 Annotation of DMRs

The pipeline also annotates all the hypo/hyper-DMRs to the genomic regions (TSS, TES, gene body, intergenic, 5′ distance and any given region like enhancers). As a result, lists of DMRs are categorically exported for each region with a corresponding P-value for each DMR. Moreover, a list of gene names having DMRs associated to their TSS, TES or gene body is also exported.

### 2.5 Visualization and statistical summary of results

Three main figures in the results provide a statistical summary of the genome-wide methylation status: 1) relative density of 5mC/5hmC (e.g., methylation levels >1 and >0.5 for HMST-Seq [11] and WGBS/RRBS data, respectively) in defined genomic regions (e.g, TSS, TES, gene body, enhancer, 5′ distance, and intergenic regions) or genome-wide; 2) percentage of hyper-/hypomethylated DMRs in TSS, TES, gene body, 5′ distance, and intergenic regions; 3) a genomic average of 5mC/5hmC levels in TSS-Gene-TES regions or enhancer regions. To plot the genomic average of 5mC and 5hmC levels in TSS, TES, gene body or enhancer regions, the package maps MRs of these regions to a new uniform range that equalizes all genes' length [31]. Here, one-dimensional nearest-neighbour algorithm was used to interpolate methylation data when mapping the original data to a new range. Subsequently, the mean of methylation levels of all genes was calculated in the new equalized range, where a centred moving average method is used to smooth the data as follows:

$${\mathrm{MA}}_{t}=\frac{1}{n}\sum_{i=-n/2}^{n/2}A_{i}$$

Here, *n* is the window size, and A*_i_* is the data point at the *i*th position. The window size is the number of observations used for calculating the statistic. In order to reduce noise when plotting the genome-wide average of methylation profiles, a one-dimensional Gaussian filter [35] is applied to further smooth the mean data before plotting them in TSS, gene body and TES regions in a single plot. The effect of the data smoothing is illustrated in SFig. 4. The genome-wide average methylation level of enhancers can be plotted in a similar way. The pipeline also exports the plot data for external visualization and analysis by users. For more information of implementation, package comparison and performance (e.g., CPU hours and memory consumption) please refer to (http://urn.nb.no/URN:NBN:no-76419).

### 2.6 Methylation data

HMST-Seq-Analyzer can handle two types of data: methylation and hydroxymethylation, from three different DNA sequencing approaches (e.g., HMST-Seq, WGBS, and RRBS). There are two types of methylation input data: 1) a bed formatted file containing methylation percentage per base, used for WGBS or RRBS data. 2) a TSV file containing normalized tag count per base from 3 libraries of HMST-seq data. The pipeline has been tested successfully in CpG, CHG, and CHH methylation from WGBS, RRBS, and HMST-seq. For WGBS, Human lymphoblastoid cell line (GM12878) and human embryonic stem cell line (H1) from ENCODE data sets are used as test and control set, respectively [9]. For RRBS, TET knockout mice data was used [36]. HMST-seq data was acquired from a study conducted on two hepato cellular carcinoma (HCC) cell lines (97L and LM6 cells), and a non-HCC sample [18].

### 2.7 Gene annotation

As DMR detection will be spanned around annotated genomic regions, gene annotation information is needed. The reference genome file is a simple tab separated text file containing the chromosome number and the size of respective chromosome, which were prepared according to assembly and species of the input samples (e.g., hg19 and mm10 chromosome size information [25]). If a bed formatted enhancer position file is available, then the pipeline can map DMRs to enhancer regions. This enhancer option can be used to map DMRs across any other regions as well e.g. CpG islands.

### 2.8 Computational efficiency of HMST-Seq-Analyzer

In order to make the pipeline suitable for large data sets, HMST-Seq-Analyzer modules *Find MRs, Preparation for DMR Search,* and *DMR Search* are optimized for parallel computation specifically. The input argument *-p* is used to define the number of CPUs a user wants to use. As a result, the corresponding task is automatically parallelized either on a single multi-core machine, or on a high-performance computer. HMST-Seq-Analyzer was tested on both a small and a large dataset to evaluate the efficiency. For small data set (~4.5 MB), chromosome 1 from a HMST-Seq mouse data was used with 70,201 and 71,646 tag counts for the KO and WT samples, respectively. For a large data set (~66 MB), the whole HMST-Seq mouse (20 chromosome) data set was used with KO (1037346 tag counts) and WT (1054613 tag counts) condition sample, respectively. The test was run on 5 CPUs with 4G memory on SAGA computer cluster at Norwegian University of Science and Technology.

## 3 Results and discussion

### 3.1 Differential methylation analysis of HMST-Seq data

**Fig. 2:**
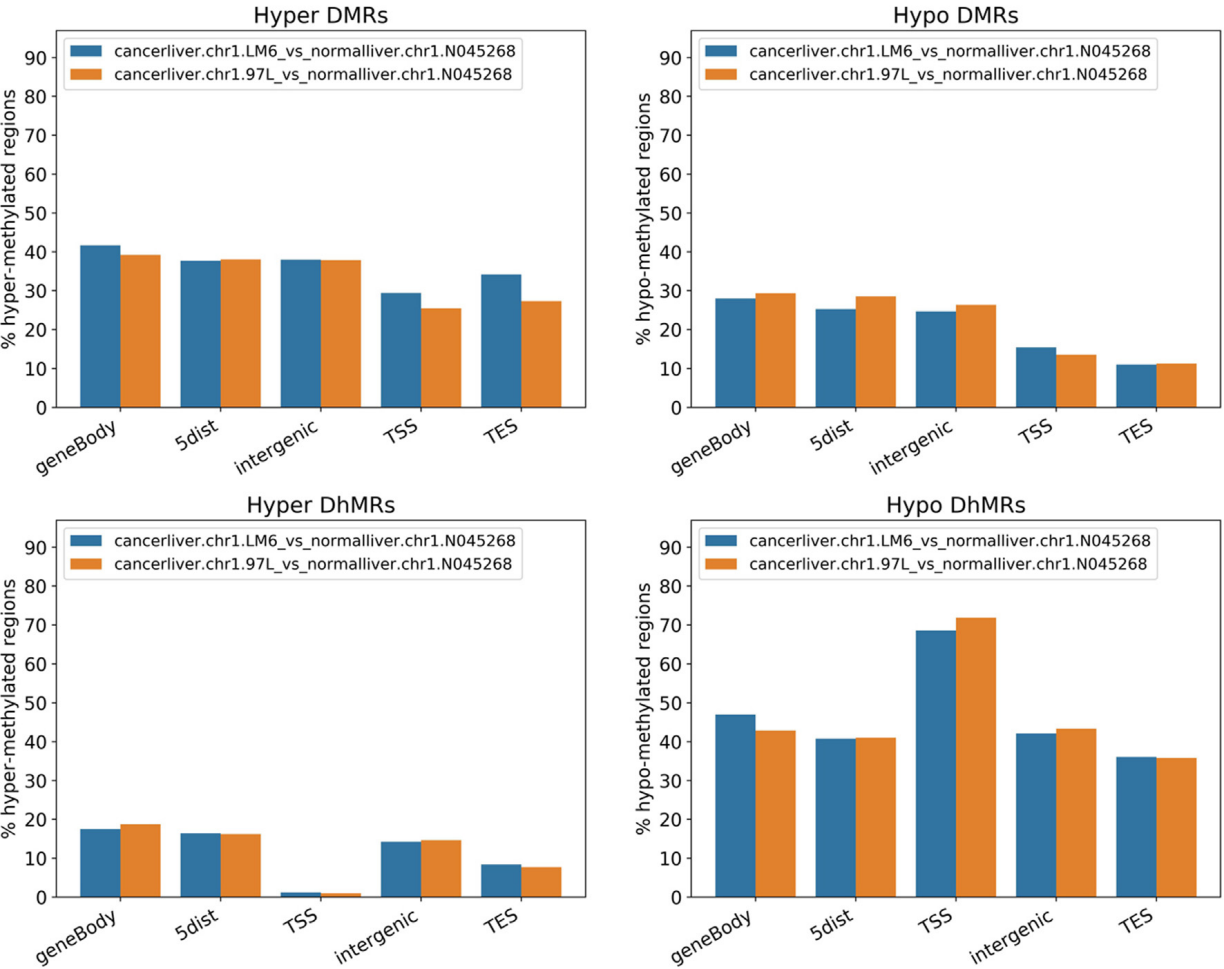
Distribution of differentially methylated (or hydromethylated) regions (DMRs/DhMRs) between the two HCC cell lines (97L and LM6) and a non-HCC sample (NO45268) in five types of genomic regions. HMST-Seq-Analyzer was applied on publicly available HMST-Seq data of three samples (two liver cancer cell lines and one normal liver sample) simultaneously. Hyper/Hypo DMRs/DhMRs between the two liver cancer samples and one normal sample were identified by the pipeline automatically. The distribution of these DMRs/DhMRs were mapped to five types of genomic regions (gene body, 5′ distance region, intergenic region, TSS, and TES) by the HMST-Seq-Analyzer. Here, the 5′ distance region is defined as the upstream of TSS from 10 Kb to 100 Kb, and the intergenic regions are genomic regions excluding gene body, TSS and TES which with the minimum and maximum length 2 Kb and 100 Kb, respectively. There is the same changing trend in each MR before using Kolmogorov-Smirnov test to predict the DMRs/DhMRs.

**Fig. 3:**
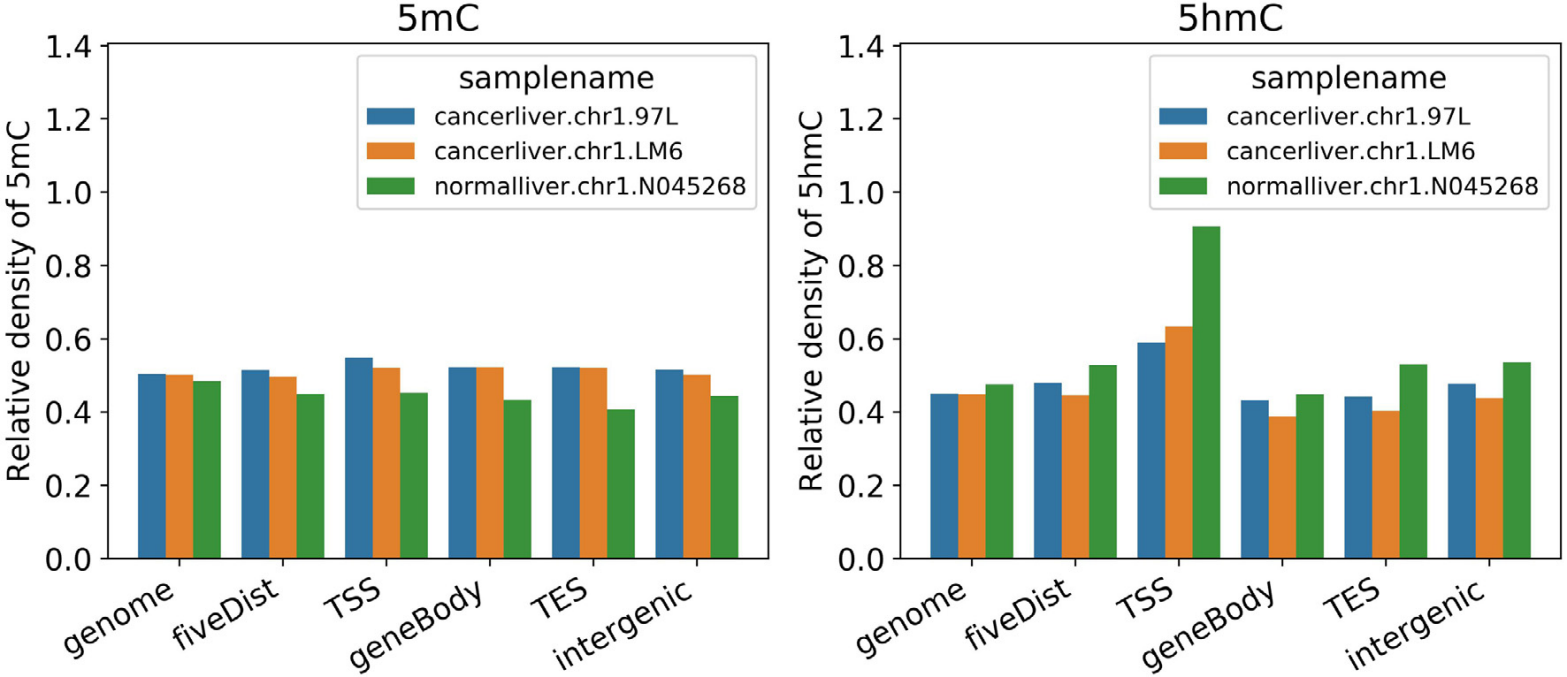
The relative density of significantly modified 5mC (or 5hmC) sites in the two HCC cell lines (97L and LM6) and one non-HCC sample, within the five types of genomic regions. HMST-Seq-Analyzer was applied on public available HMST-Seq data of three samples (two liver cancer cell lines and one normal liver sample) simultaneously. Significantly modified (methylation/hydromethylation levels > 1) 5mC/5hmC sites in the three samples were identified and their relative density in five types of genomic regions (gene body, 5′ distance region, intergenic region, TSS, and TES) was calculated by the pipeline automatically. The 5′ distance region is defined as the upstream of TSS from 10 Kb to 100 Kb. The intergenic regions are regions excluding gene body, TSS and TES, which with the minimum and maximum length 2 Kb and 100 Kb, respectively.

**Fig. 4:**
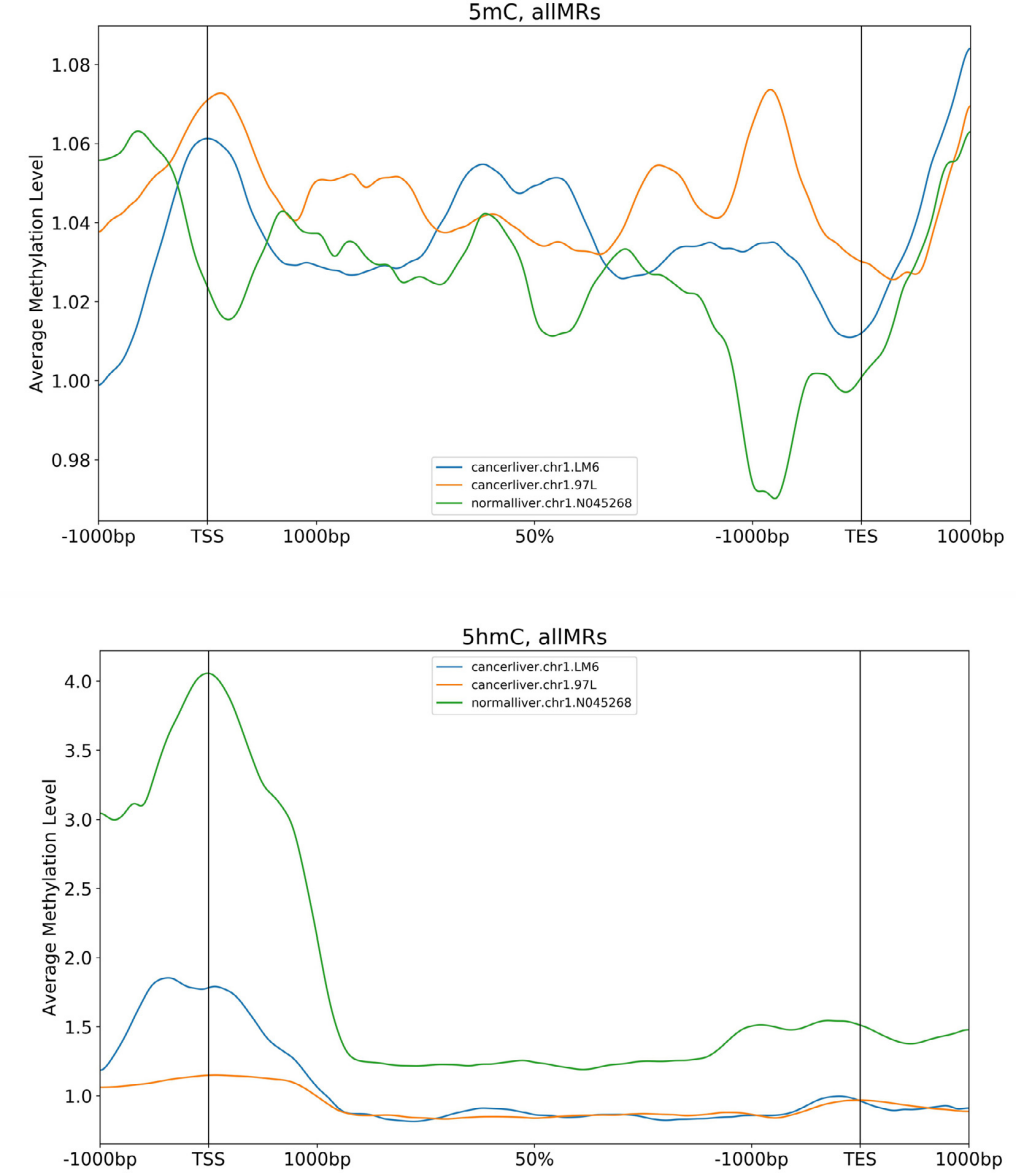
The distribution of genome-wide average of 5mC *(or 5hmC)* levels in TSS-Gene-TES regions for both HCC cell lines (97L and LM6) and non-HCC sample. HMST-Seq-Analyzer was applied on publicly available HMST-Seq data of three samples (two liver cancer cell lines and one normal liver sample) simultaneously. Methylated regions (MRs) of 5mC and 5hmC in three different samples are identified by the pipeline in TSS, TES, and gene body regions, respectively. A genomic average of 5mC/5hmC levels in all TSS-Gene-TES regions are calculated, where a centred moving average method is applied to smooth the data. Subsequently, one-dimensional Gaussian filter is used to reduce noise in smoothed mean data before plotting the genome-wide average of methylation levels in TSS-Gene-TES region.

Numerous studies have reported a role of altered methylation of tumor suppressor genes in the pathogenesis of human hepatocellular carcinoma (HCC) [37]. In addition, 5-hmC levels are reported to be lower in HCC tissues in comparison to non-tumor tissues [38]. Simultaneous inspection of 5mC and 5hmC levels in HCC can reveal important characteristics of epigenetic alterations in HCC. In a study by F. Gao *et al.*, HMST-seq was performed on two HCC cell lines (97L and LM6 cells), and a non-HCC sample [18]. In this work, HMST-Seq-Analyzer with default parameters on chromosome 1 of this dataset was run and tag counts were aligned to the same human reference genome (hg19). All results and figures were generated by a single run of HMST-Seq-Analyzer on both 5mC and 5hmC data simultaneously. Fig. 2 shows that there is not a strong difference between hyper-DMRs and hypo-DMRs distributions in different genomic regions. However, a lower number of hyper-DhMRs but higher number of hypo-DhMRs in HCC cell lines as compared to the non-HCC cell line (lower panel of Fig. 2), especially in TSS regions are clearly seen. Fig. 3 suggests that the number of significantly modified 5mC and 5hmC sites are lower and higher for the non-HCC sample in all genomic regions (especially in TSS) than that of HCC samples, respectively. In Fig. 4, the 5mC levels around TSS seem to be slightly higher in the HCC samples than in the non-HCC sample. In contrast, 5hmC levels are increased more than twofold around TSS in non-HCC compared to HCC samples. Another study also found uneven distribution of 5mC and 5hmC in TSS regions of tumor samples as compared to non-tumor samples [39]. They observed overall high 5mC levels and lower 5hmC levels in tumor tissues. These results are also in agreement with the results predicted by HMST-Seq-Analyzer as shown in Fig. 2, where ~70% of MRs in the TSS region are DhMRs while only ~10% are DMRs. Thus, our new pipeline reproduces the results of the original publication in a single run, which simplifies the data interpretation. More interesting results can be found if the pipeline is run on the complete data set. For example, significantly differentially methylated genes for hepatocellular carcinoma can be drawn out from the gene list exported by the proposed tool.

### 3.2 A comparison of HMST-Seq-Analyzer and MethylKit in analysing differentially methylated CpG sites

Currently, there is lack of publicly available tools for analysing HMST-Seq data. To evaluate the robustness of differential methylation analysis in HMST-Seq-Analyzer, results are compared to a popular tool - MethylKit, which uses Fisher’s Exact Test (FET) to identify differentially methylated CpG sites (DMCs) [21] in WGBS or RRBS data.

#### 3.2.1 HMST-Seq Analyzer recovers MethylKit DMCs in WGBS

**Fig. 5:**
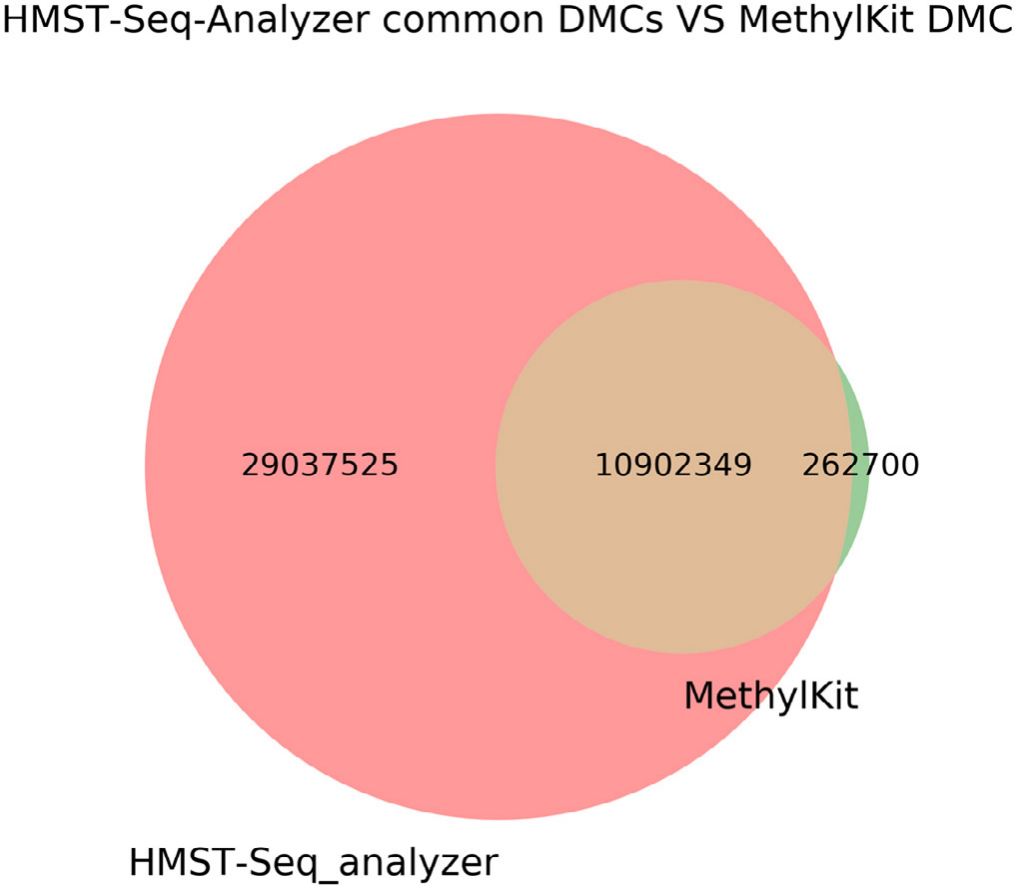
Whole-genome DMC comparison for WGBS (HMST-Seq-Analyzer VS MethylKit). Number of genome-wide DMCs predicted by HMST-Seq and MethylKit are shown in figure. Unique DMCs predicted by HMST-Seq-Analyzer are represented in Pink, by MethylKit in green and overlapping in brown, respectively. Both HMST-Seq-Analyzer and MethylKit were run in default parameters. Here, ~98% of the DMCs identified by MethylKit are overlapping with DMCs predicted by HMST-Seq-Analyzer. (For interpretation of the references to colour in this figure legend, the reader is referred to the web version of this article.)

Here, a WGBS data set for human genome was gathered from the ENCODE project. Human lymphoblastoid cell line (GM12878) and human embryonic stem cell line (H1) are used as test and control set, respectively [9]. Raw sequencing data set was mapped to hg38. Due to enormity of WGBS data, it was split chromosome wise for analysis on both tools. Results were later combined for whole-genome level analysis. The default parameters of HMST-Seq-Analyzer were used, except for shortening the 5′-Distance regions between 10 kbp to 50 kbp from TSS, in order to reduce the computation time. For MethylKit, a q-value < 0.05 with minimum percent methylation difference cut-off of 25% are used to identify differentially methylated cytosines (DMCs). For HMST-Seq-Analyzer, we extracted DMCs from our predicted DMRs before comparing them to those provided by MethylKit. In total, HMST-Seq-Analyzer and MethylKit reported 47 million and 11 million DMCs sites, respectively. HMST-Seq-Analyzer recovered 97.6% of the mCs reported by MethylKit (Fig. 5). While MethylKit reports only individual DMCs, HMST-Seq-Analyzer gives much more information along recovering almost all the DMCs reported by MethylKit.

#### 3.2.2 A comparison of HMST-Seq-Analyzer and MethylKit in RRBS

In this comparison, RRBS data was obtained from experiments with TET knockout mice [36]. Both HMST-Seq-Analyzer and MethylKit were applied on the same data, to detect differential methylation events between TET1 and TET2 double knockout and wild type mice. In terms of the number of CpGs per kb, RRBS has much sparse methylation sites than that in WGBS, hence we performed whole-genome differential methylation detection in a single batch. For MethylKit, the default parameters were used for detecting genome-wide DMCs. For example, a q-value < 0.05 with minimum percent methylation difference cut-off of 25% was used to identify DMCs as used in the source publication [36]. For HMST-Seq-Analyzer, genome-wide DMCs were extracted from DMRs reported by the package before comparing with the MethylKit results, where the number of minimum consecutive sites is 2, adjacency is 200 bp, the same methylation changing trend, and T-test were used in DMR analysis. HMST-Seq-Analyzer reported 4242 DMCs in its DMRs, while MethylKit reported 15,756 DMCs in total, in which ~48% of the DMCs obtained from HMST-Seq-Analyzer overlap with MethylKit DMCs (SFig. 5). If the same methylation changing trend is disabled in HMST-Seq-Analyzer, then the percentage of overlapping DMCs drops slightly to 46% (SFig. 6). Though half of the predicted DMCs by HMST-Seq-Analyzer are recovered by MethylKit, HMST-Seq-Analyzer gives much fewer DMCs than MethylKit. This is mainly caused by the two packages using entirely different methods to predict DMCs. HMST-Seq-Analyzer has a restriction of adjacency in nearby CpGs and of minimum number of CpGs in MR. Moreover, DMCs are extracted from the predicted DMRs by using T-test. On the contrary, MethylKit is designed to report DMCs only but does not consider the spatial distribution of CpGs in a MR, and the significance of DMCs is tested on individual CpG directly based on Fishers’ exact test.

In further analysis, a correlation between the percentage of overlapping DMCs and the change of adjacency in HMST-Seq-Analyzer is studied. DMCs predicted by HMST-Seq-Analyzer with different adjacencies are compared against to the prediction of MethylKit with default parameters. The result in SFig. 7 suggests that a smaller adjacency in HMST-Seq-Analyzer gives a higher percentage of overlap between the two methods. For example, the percentage of overlapping DMCs is increased from about 41% to 51%, when the CpG adjacency is reduced from 3000 bp to 100 bp. Therefore, the discrepancy of predictions between the two packages is expected, which is mainly due to the difference in prediction method such as the restriction of spatial distribution for nearby CpGs in HMST-Seq-Analyzer. A list of optimal parameters for HMST-Seq-Analyzer is provided in the package. For RRBS data analysis, it is recommended to use adjacency of 200 bp (*−a* = 200), the same methylation changing trend (−*isST = 1*), a minimum number of 2 consecutive sites (*−mc1* = 2, −*mc2* = 2, −mc3 = 2) and T-test for DMR prediction. For WGBS data, we suggest to use window bin size 200 bp (−W = yes, −a = 200), a minimum of 3 consecutive sites (*−mc1* = 3, −*mc2* = 5, −*mc3* = 3) and T-test for detecting DMRs. In case of HMST-seq, the default parameters are adjacency 2 kb, a minimum of 3 consecutive sites (*−mc1* = 3, *mc3* = 3) and 5 (*−mc2* = 5) for TSS/TES/enhancer and gene body/5′ distance/intergenic regions, respectively. More information of optimal parameter selection for HMST-Seq-Analyzer is included in the package.

#### 3.2.3 HMST-Seq Analyzer captures important information that is missed by MethylKit

**Fig. 6:**
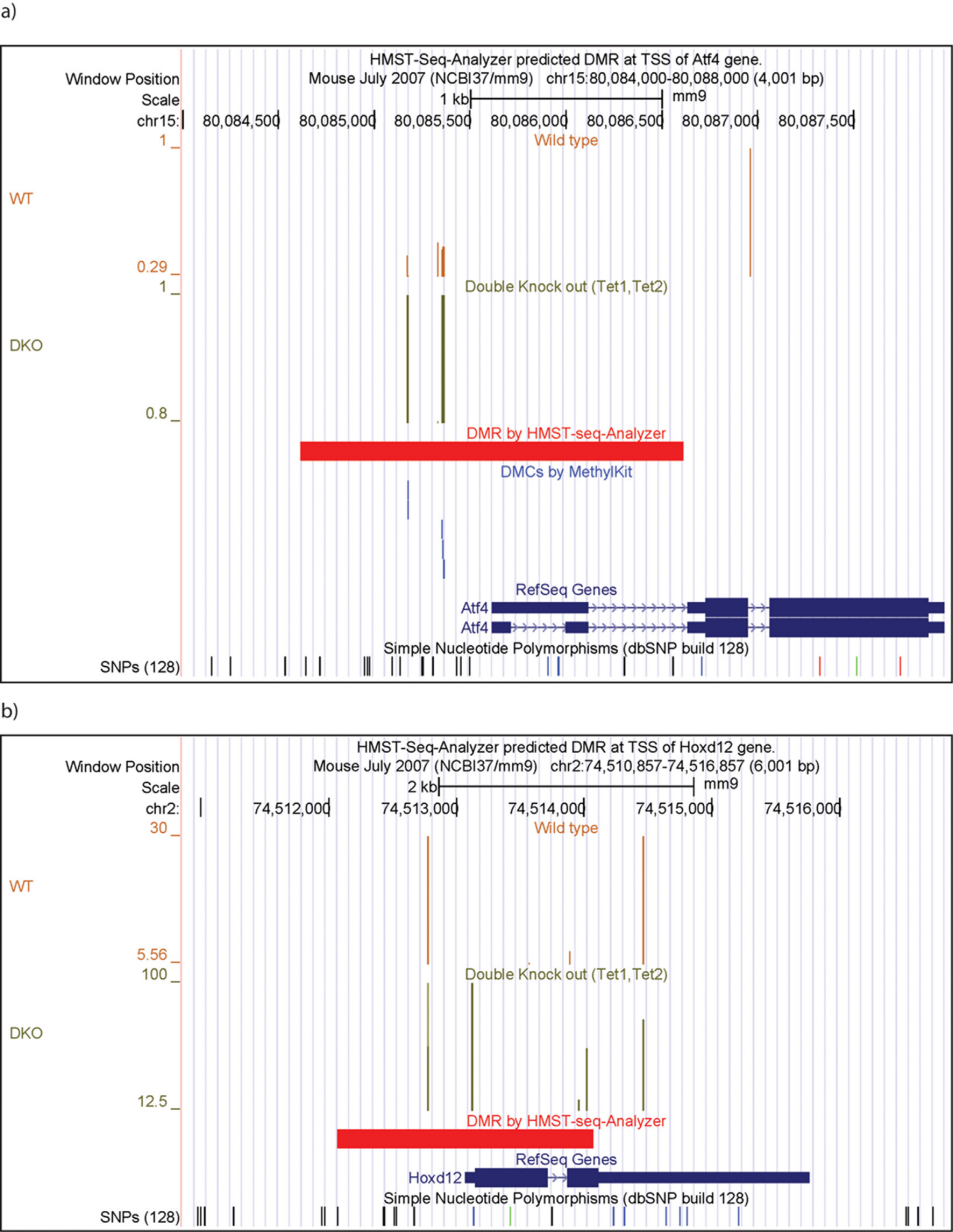
HMST-Seq-Analyzer predicted DMR at TSS of multiple genes. Panels a and b are for genes Atf4 and Hoxd12 respectively. The first track represents the methylation level at mCs from wild type (WT) by orange bars, with the height of each vertical bar representing percentage of methylation. The second track represents the methylation level at mCs from TET1, TET2 double knock out (DKO) in green. The third track represents DMRs detected by HMST-Seq-Analyzer in red. The fourth track presents DMCs predicted by MethylKit in blue. The fifth track depicts the gene in dark blue. No DMCs were predicted by MethylKit for Hoxd12, hence, the track for methylkit is not included in pancel b. (For interpretation of the references to colour in this figure legend, the reader is referred to the web version of this article.)

To further evaluate the results between the two tools, we focus on the promoter regions (±1 Kb to TSS) from the first round of RRBS analysis with default parameters. HMST-Seq-Analyzer reported 291 genes having more than two DMCs in the promoter regions (STable 1). For MethylKit, it reported a total of 3938 genes with DMCs in the promoters but the majority (61%) of them had only one DMC (SFig. 8). There are 91 genes overlapping between the MethylKit and HMST-Seq-Analyzer results (STable 2). For instance, Atf4 which is a transcription factor, was reported as having DMCs occurring at the promoter region by both tools (Fig. 6a). Because of the wide difference in results from the two tools, it is interesting to perform functional gene annotation on the two gene lists. DAVID tools was used for this analysis [40]. GO results (P-value < 0.05) of the genes predicted exclusively by HMST-Seq-Analyzer (~200) and MethylKit (e.g., the top 200) are presented in STables 3 and 4, respectively. The highest number of genes (20) predicted by HMST-Seq-Analyzer are involved in organismal development (STable 3). This was also reported in the original study that deletion of TET proteins impairs differentiation in embryonic stem cells, which is clearly linked to organismal development [36]. Since there were too many MethylKit predicted genes (>3800) with DMCs in promoters to give a meaningful gene enrichment test, the top 200 differentially methylated genes (on the basis of the P-value from MethylKit) were selected for functional gene annotation (STable 4). Here, genes were reported to be involved in 15 different processes, with the highest number of them being linked to transcription (GO:0006351, 23 genes) and negative regulation of transcription (GO:0000122, 14 genes). However, the original study reports [36] that while the TET protein deletion did alter the DNA methylation at some promoters, it did not correlate with the corresponding gene expression changes. Moreover, percentage of genes with only 1 DMC in the promoter further increases to ~70% in the top 200 results of MethylKit (SFig. 9). A similar result (STable 5) was obtained when the same analysis was repeated with the top 500 genes from the MethylKit prediction.

**Fig. 7:**
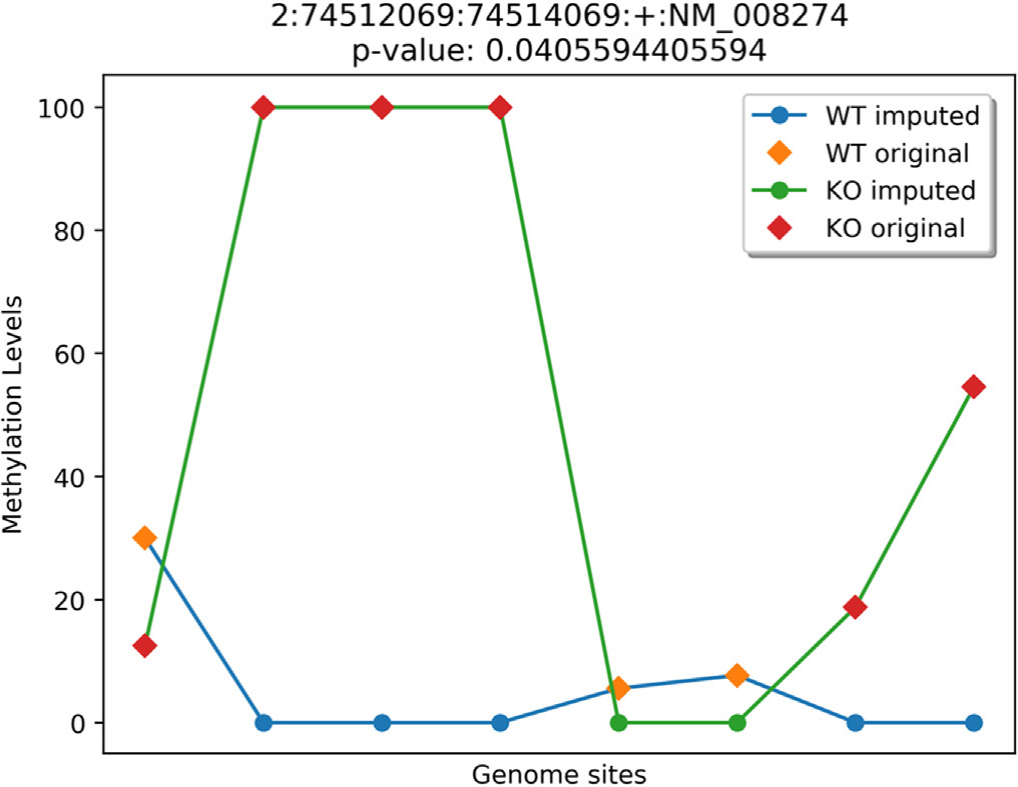
Distribution of Methylated Cytosines in HOXD12 DMR predicted by HMST-Seq-Analyzer. The heading of the figure provides information about the DMR in format “chromosome number:start position:end position:strand:gene identifier” followed by the p-values. Circles represent imputed methylation values at each methylated site (blue for wild type/control and green for knockout/case). Diamonds represent original methylation value (orange for wildtype/control and red for knockout/case). (For interpretation of the references to colour in this figure legend, the reader is referred to the web version of this article.)

Literature evidences of a few genes predicted by HMST-Seq-Analyzer but missed by MethylKit are provided in Table 1, where the gene Hoxd12 is particularly interesting because it is directly targeted by TET1 [41]. TET1 is involved in active demethylation and decreased recruitment of TET1 to the HOXD12 gene results in increased methylation of HOXD12. HOXD12 is also reported to have hypermethylated CpG as a potential marker for stage 1 lung squamous cell carcinoma [42]. Hence, HOXD12 must specifically be reported as differentially methylated in the TET knockout data. While MethylKit failed to identify HOXD12 as differentially methylated, HMST-Seq-Analyzer not only reported it as differentially methylated but also correctly categorized it as hypermethylated as expected in absence of TET proteins (Fig. 6b). In Fig. 7, the distribution of mCs in this DMR was plotted by HMST-Seq-Analyzer, where the methylation levels from TET knock out and wild type are different. For the sites 2–4 in Fig. 7, the WT sample provided no methylation information for the highly methylated sites in KO. MethylKit relies on single base pair differential methylation analysis that discards such sites with missing values, which failed to capture the DMCs in promoter of HOXD12. It is reasonable to assume that WT mCs at sites 2–4 experienced demethylation in presence of TET proteins and consider them in analysis, instead of assuming that the three consecutive sites experienced experimental error and ignoring them. This argument is more plausible especially when mCs sites 2–4 are not partially but fully methylated in the KO sample.

**Table 1:** Literature evidence for genes predicted by HMST-Seq-Analyzer but missed by MethylKit in differentially methylated CpG sites analysis.

Gene Name	Comments	References
Mael	1) hypomethylated in colorectal cancer. 2) Hypermethylation of MAEL promoter in infertile men. 3) Mael promoter methylation levels are all increased in tet1 tet2 mutants.	[48], [49]
Hoxd12	1) recovered as differentially methylated by swDMR (another tool). 2) Hoxd12 is targeted by TET1 and decreased recruitment of Tet1 on HOXD12 gene results in increased methylation. 3) hypermethylated CpGs in HOXD12 gene reported as potential marker for stage 1 lung squamous cell carcinoma.	[30], [41], [42], [30], [42], [50]
Dazl	1) promoter is usually methylated (it’s usually expressed in germ cells, so unmethylated in germ cells only)	[51]
Shank2	1) hyper methylated in prostate cancer	[52]
Rn45s	1) hypo methylated in mice with high maternal folic acid.	[53]

To identify differentially methylated CpG sites (DMCs), HMST-Seq-Analyzer and MethhylKit were applied on the same RRBS data of TET knockout mice experiments. There are 291 and 3938 genes that contain DMCs in the promoters (+/−1Kb) based on the prediction of HMST-Seq-Analyzer and MethylKit, respectively. Selected genes with literature support are listed here.

Because relatively low coverage per site increases the sampling variation [43], variation at single sites is usually greater than that of a region. Translating variation on a single CpG site to a differential methylation event can thus be misleading. By imputing the missing values and considering the overall methylation pattern of adjacent CpG sites, HMST-Seq-Analyzer discovers important differentially methylated genes that are completely missed by MethylKit. Therefore, there are two main reasons to the large difference in the number of genes with DMCs in promoters reported by the two tools. MethylKit predicts individual DMCs and then reports the nearest feature (a gene's promoter), hence it reports a huge number of promoters where majority of them (~61%; SFig. 8) have only one DMC. Labelling whole promoter to be differentially methylated on basis of one DMC can be misleading. On the other hand, HMST-Seq-Analyzer has stringency parameters (e.g., ≥2 DMC at a promoter), which filter out multiple standalone methylated sites. This reduces the total number of promoters reported at the end of analysis, but provides significant results as every DMR would have at least two DMCs. In summary, DMCs predicted by HMST-Seq-Analyzer capture more significant biological events than MethylKit.

### 3.3 A comparison of HMST-Seq-Analyzer with more packages for analysing differentially methylated regions

Though no public standalone tool is available for HMST-Seq data analysis, there are several R packages (e.g., BSmooth [22], MethylSig [23], and MethylKit [21]) that can be used to detect DMR in WGBS experiments. We used WGBS data for two human cell lines (H1 and IMR90; each cell line with replicate) that included in BSmooth package, to evaluate the performance of DMR prediction in HMST-Seq-Analyzer and three R packages (BSmooth, MethylSig, MethylKit). First, WGBS datasets of the two human cells of the original publication [44] were downloaded using R scripts in BSmooth. CpG sites with coverage less than 4 are removed. Then, default settings of each program were used in computation. For example, smoothed methylation levels by the BSmooth function were used in both BSmooth and MethylSig, but raw methylation levels were inputted into MethylKit and HMST-Seq-Analyzer. In each program, missing value imputation and data interpretation were based on the default settings. All calculations were performed on a computer node of the Sigma2 Saga computer cluster at the Norwegian University of Science and Technology.

In selected four packages, the definition of MR is different. Both MethylKit and MethylSig consider MR as either tiling windows (25 bp or default 200 bp) or a set of predefined regions (e.g., promoters). BSmooth defines MR by a maximum gap (default is 300 bp) between adjacent CpG sites and a minimum number (default is 3) of CpG sites in a MR. This is similar to the MR search strategy in HMST-Seq-Analyzer (e.g., default maximum distance between adjacent CpG sites is 2000 bp and minimum 3 CpG sites in a MR). To identify DMRs between the two human cell lines (e.g., H1 versus IMR90), MethylKit, MethylSig, BSmooth and HMST-Seq-Analyzer apply logistic regression test, Beta-binomial test, T-statistics and T-test on MRs with the same changing trend, respectively. Since only HMST-Seq-Analyzer considers both DMR finding and gene annotation simultaneously when searching for DMRs, the time usage for DMR finding and gene annotation was separated in the evaluation. Results of the performance comparison on human chromosome 22 are shown in Table 2, where the detected DMRs from each method were sorted and merged by BEDTools [45] before counting their overlap to the predictions from HMST-Seq-Analyzer. In summary, ~80% of DMRs detected in each R package are recovered by HMST-Seq-Analyzer, regardless of the tiling window size (e.g., either 25 bp or 200 bp) that was used in MethylKit and MethylSig. BSmooth has the highest percentage (~86%) of overlapping DMRs with HMST-Seq-Analyzer. A median size of the predicted DMRs from HMST-Seq-Analyzer (~10 Kb) is much longer than that of the other packages (e.g., ~200 to 400 bp). Though HMST-Seq-Analyzer used more running time (~400 s) than most of R packages (e.g., ~60 to 533 s), around 75% of its wall time were used in the gene annotation. Similar results were also obtained from both a median size human chromosome (chr17; STable 6) and a large human chromosome (chr1; STable 7), when the same evaluation was performed. Overall, the predicted DMRs from HMST-Seq-Analyzer is robust against those from the three R packages in WGBS data analysis (e.g overlap to ~80% of BSmooth and ~70% of MethylSig/MethylKit predictions). However, the computational efficiency of HMST-Seq-Analyzer needs further improvement by optimizing the gene annotation step during the DMR finding. One advantage of HMST-Seq-Analyzer over the other tools is that it saves time for post processing. A user already knows that every DMR will contain a minimum number of methylated sites and will be annotated to a genomic feature.

**Table 2:** A comparison of HMST-Seq-Analyzer and three other packages in differentially methylated region analysis (chr22).

	DMRs	Overlapping	Percentage (%)	Length (bp)	Gene annotation (second)	DMR finding (second)	Total (second)
HMST-Seq-Analyzer	1107	1107	100	9768	308	86	394
BSmooth	1105	953	86	379	NA	60	60
MethylSig	34,347	27,816	81	200	NA	168	168
MethylKit	12,460	9865	79	200	NA	87	87
MethylSig	50,597	41,182	81	25	NA	533	533
MethylKit	16,683	13,371	80	25	NA	115	115

HMST-Seq-Analyzer, BSmooth, MethylSig, and MethylKit were applied on the same WGBS data to identify differentially methylated regions (DMRs) between human H1 and IMR90 cells, respectively. Here, each cell line has replicated experiments and only chromosome 22 is used in the evaluation for all programs. MethylKit and MethylSig are tested in two different lengths of window size (25 bp and 200 bp). In the table, DMRs represents the number of DMRs detected by the package. Overlapping and Percentage are the number of and the percentage of DMRs that are overlapping with the DMRs from HMST-Seq-Analyzer, respectively. Length is the median length of DMRs. DMR finding, Gene annotation and Total are wall time (seconds) used in each step, respectively.

## 4 Conclusion

We present a new Python package called HMST-Seq-Analyzer for differential methylation and hydroxymethylation analysis, that identifies and annotates genome-wide differentially methylated regions (DMRs) by using DNA sequencing data. Though it is optimized for HMST-Seq data, the tool is highly flexible and is able to analyse other popular types of DNA methylation sequencing data such as WGBS and RRBS. HMST-Seq-Analyzer takes as an input either library tag counts of HMST-Seq or methylation percentage per base in the case of WGBS/RRBS for DMR detection. Regardless of pre- and postprocessing steps, this pipeline can be used independently to detect DMRs. Gene annotation of discovered DMRs is performed automatically at the end of the pipeline, summary statistics of methylation distributions in various genomic regions (e.g., TSS, TES, gene body, and 5′ distance region) are illustrated in graphs, and the average of methylation levels spanning gene regions such as TSS-gene-TES or enhancer regions are also provided. The package is able to deal with very huge data sets such as genome-wide methylation profiles of CpG, CHG, or CHH methylation from WGBS data, because of the parallel implementation of the MR/DMR search algorithms. The final results exported by the pipeline (e.g. gene annotated hyper/hypo DMRs) are ready for biologists to be used for further detailed investigation.

HMST-Seq-Analyzer is written in Python and is publicly available. It is a command line tool and tested on both macOS and Linux operating systems. For small or medium sized data sets (e.g., RRBS/HMST-Seq), it can be run on desktop PCs. However, for big data (e.g., genome-wide CpG/CHG/CHH from WGBS), it is preferable to run the pipeline on high performance computers with parallel computation. The package is able to process both human and mouse data, as well as to the other species if the corresponding reference genomes are available and with the same format as our provided human/mouse genome. For convenience, human (hg 19, hg38) and mouse (mm10) genome annotation and chromosome size files are also included in the package. Though the current pipeline is robust in differential methylation analysis when compared to three popular R packages (overlapping with ~80% BSmooth and ~70% MethylKit/MethylSig predicted DMRs), it is slow in processing of gene annotation during the DMR finding. Especially, when multiple groups, conditions or replicates are considered in DMR prediction. In future, we aim to overcome this limitation by developing new methods suitable for fast gene annotation and further optimize its speed for very large data such as genome-wide non-CpG methylation. We will also consider adapting the software for integration into Galaxy [46], CyVerse Discovery Environment [47], or similar platforms, as well as enabling export of results for visualization with the UCSC Genome browser [25] or other tools.

## CRediT authorship contribution statement

**Amna Farooq:** Software, Validation, Formal analysis, Visualization, Writing - original draft. **Sindre Grønmyr:** Software, Visualization, Validation, Formal analysis. **Omer Ali:** Validation, Formal analysis, Writing - review & editing. **Torbjørn Rognes:** Validation, Writing - review & editing. **Katja Scheffler:** Validation, Writing - review & editing. **Magnar Bjørås:** Validation, Writing - review & editing. **Junbai Wang:** Conceptualization, Methodology, Software, Data curation, Writing - review & editing, Visualization, Investigation, Formal analysis, Supervision, Validation, Project administration, Funding acquisition.

## Declaration of Competing Interest

The authors declare that they have no known competing financial interests or personal relationships that could have appeared to influence the work reported in this paper.
