# Supplementary material for: HMST-Seq-Analyzer: A new python tool for differential methylation and hydroxymethylation analysis in various DNA methylation sequencing data
Source: Comput Struct Biotechnol J. 2020 Oct 10;18:2877–89. doi: 10.1016/j.csbj.2020.09.038 (PMC7593523; doi:10.1016/j.csbj.2020.09.038)
Supplement: Supplementary data 1 [file mmc1.docx]

**Supplementary to “HMST-Seq-Analyzer: A New Python Tool for Differential Methylation and Hydroxy-methylation Analysis in Various DNA Methylation Sequencing Data”**

**Amna Farooq^1^, Sindre Grønmyr^1, 2^, Omer Ali^1^, Torbjørn Rognes^2,4^, Katja Scheffler^6,7^, Magnar Bjørås^3,4,5^, Junbai Wang^1*^**

1. Department of Pathology, Oslo University Hospital - Norwegian Radium Hospital, Oslo, Norway
2. Department of Informatics, University of Oslo, Oslo, Norway
3. Institute for Clinical and Molecular Medicine, Norwegian University of Science and Technology, Trondheim, Norway.
4. Department of Microbiology, Oslo University Hospital, Oslo, Norway
5. Department of Microbiology, University of Oslo, Oslo, Norway
6. Department of Neuromedicine and Movement Science and Department of Clinical and Molecular Medicine, Norwegian University of Science and Technology, Trondheim, Norway
7. Department of Neurology and Department of Laboratory Medicine, St. Olavs Hospital, Trondheim, Norway

*To whom correspondence should be addressed.

Email: junbai.wang@rr-research.no

# **Supplementary Figures**

**S Figure 1: Five genomic regions used for DMR annotation by HMST-Seq-Analyzer**

HMST-Seq-Analyzer extracts five different genomic regions on the basis of the genes provided in the reference genome. The extracted genomic regions are TSS, gene body, TES, intergenic, and 5’ distance. Panel A shows two genes and their respective TSS and TES on positive strand of the reference genome. Panel B shows the genomic regions extracted by HMST-Seq-Analyzer on basis of A. Both genes have + strands.


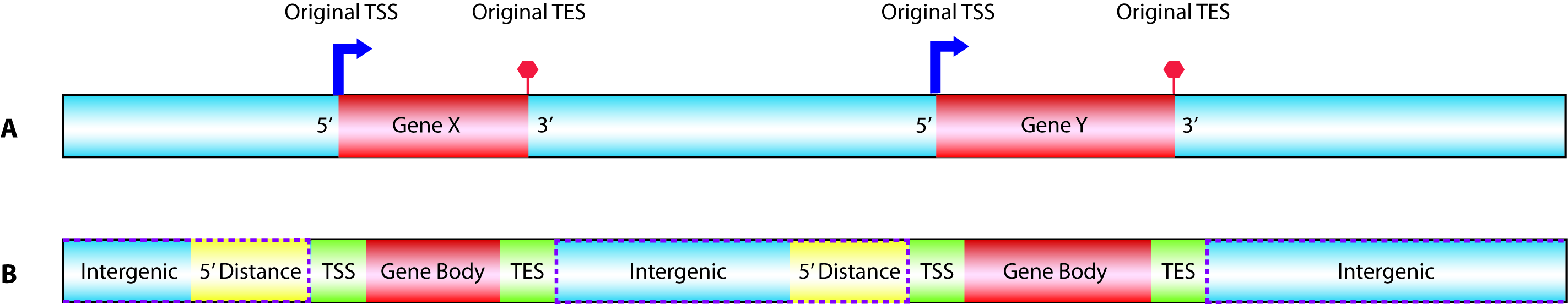


**S Figure 2: Time consumption of the DMR Search task by using three different test methods.**

Here, three different rank-sum test methods (Mranksum, Pranksum, and Rranksum) were applied on HMST-Seq liver cancer demo data (included in our package) to search for DMRs. The number of processes are varied from 1 to 10. Blue, orange, and green lines show the CPU time used by Mranksum, Pranksum, and Rranksum, respectively.


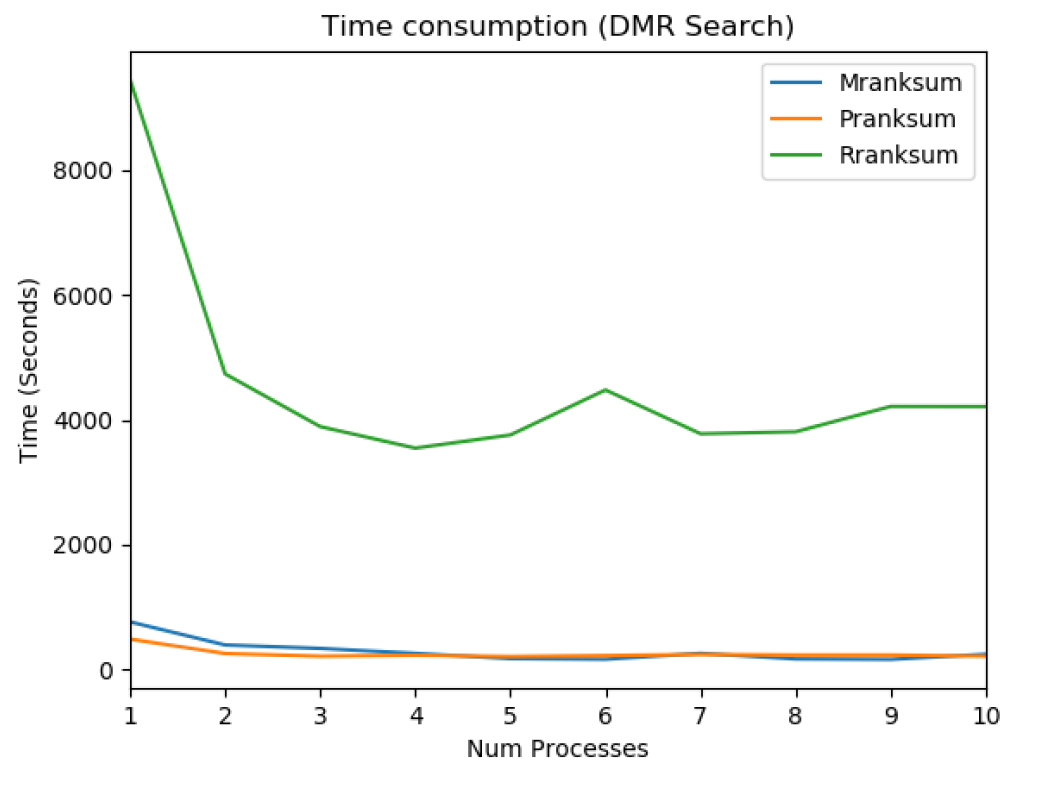


**S Figure 3: Memory consumption of the DMR Search task by using three different test methods.**

Here, three different rank-sum test methods (Mranksum, Pranksum, and Rranksum) were applied on HMST-Seq liver cancer demo data (included in our package) to search for DMRs. The number of processes are varied from 1 to 10. Blue, orange, and green lines indicate the memory consumption of Mranksum, Pranksum, and Rranksum, respectively.


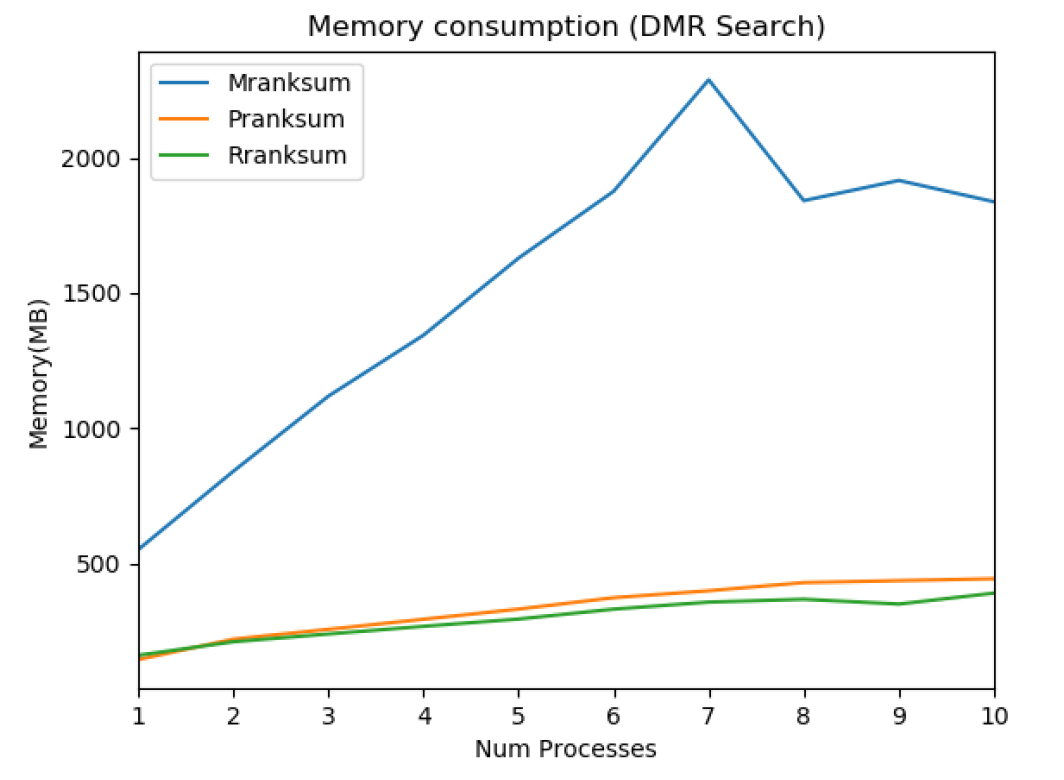


**S Figure 4: Effect of data smoothing in methylation profile plotting.**

Here, raw methylation data points, raw data smoothed by a centered moving average method, and final smoothed data by a one-dimensional Gaussian filter are shown as blue dots, orange smooth line, and red smooth line, respectively. The x-axis and the y-axis are position and methylation level, respectively.


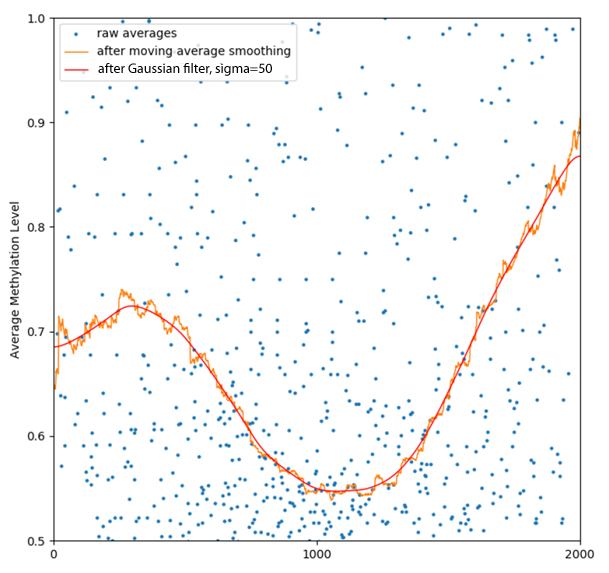


**S Figure 5: First round of DMC comparison in RRBS data analysis (HMST-Seq-Analyzer VS MethylKit)**

The number of genome-wide DMCs predicted by HMST-Seq and MethylKit are shown in figure. Unique DMCs predicted by HMST-Seq-Analyzer are shown in pink, by MethylKit in green and overlapping in brown. HMST-Seq-Analyzer was run with the following parameters for RRBS data set: adjacency (-a) = 200kb, minimum consecutive sites mc1 = 2, mc2 = 2, mc3 = 2, with the same methylation changing trend (-isST=1) and two sampled T-test in DMR detection (-T=Ttest). For MethylKit, all default parameters were used. Here, ~48% of the DMCs in DMRs predicted by HMST-Seq-Analyzer are overlapping with DMCs predicted by MethylKit.

**
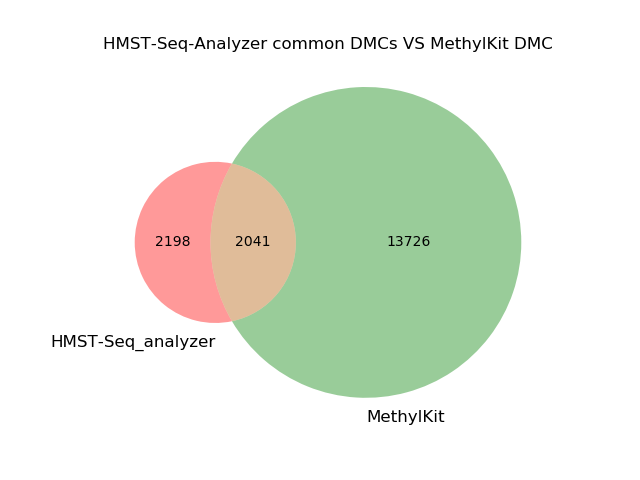
**

**S Figure 6: Second round of DMC comparison in RRBS data analysis (HMST-Seq-Analyzer VS MethylKit)**

The number of genome-wide DMCs predicted by HMST-Seq and MethylKit are shown in the figure. Unique DMCs predicted by HMST-Seq-Analyzer are shown in pink, by MethylKit in green and overlapping in brown. HMST-Seq-Analyzer was run with the following parameters for RRBS data set: adjacency (-a) = 200kb, minimum consecutive sites mc1 = 2, mc2 = 2, mc3 = 2, with mixed methylation changing trend (-isST=0) and two sampled T-test in DMR detection (-T=Ttest). For MethylKit, all default parameters were used. Here, ~46% of the DMCs in DMRs predicted by HMST-Seq-Analyzer are overlapping with DMCs predicted by MethylKit.


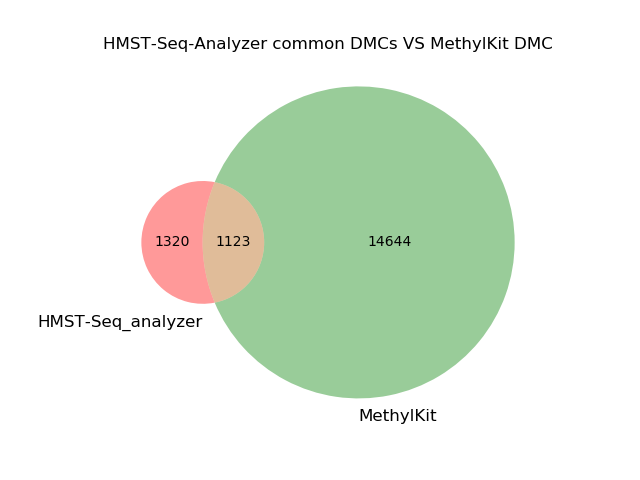


**S Figure 7: Percentage of overlapping DMCs between the HMST-Seq-Analyzer predictions and the MethylKit result.**

Figure shows the percentages of overlapping DMCs between the results of HMST-Seq-Analyzer and MethylKit. Results shown are from 5 different settings of CpG adjacency in HMST-Seq-Analyzer: (adjacency = 100bp, 200bp, 500bp, 1000bp, 3000bp). In HTMS-Seq-Analyzer, the number of methylated sites were kept to 1 for finding MRs (-mc1=1, mc2=1, mc3=1) and while finding differential methylation it was kept at 2 (-mc1=2, mc2=2, mc3=2). The same methylation changing trend (-isST=1) and T-test (-T=Ttest) was applied in the analysis. For methylKit, all default parameters were used.


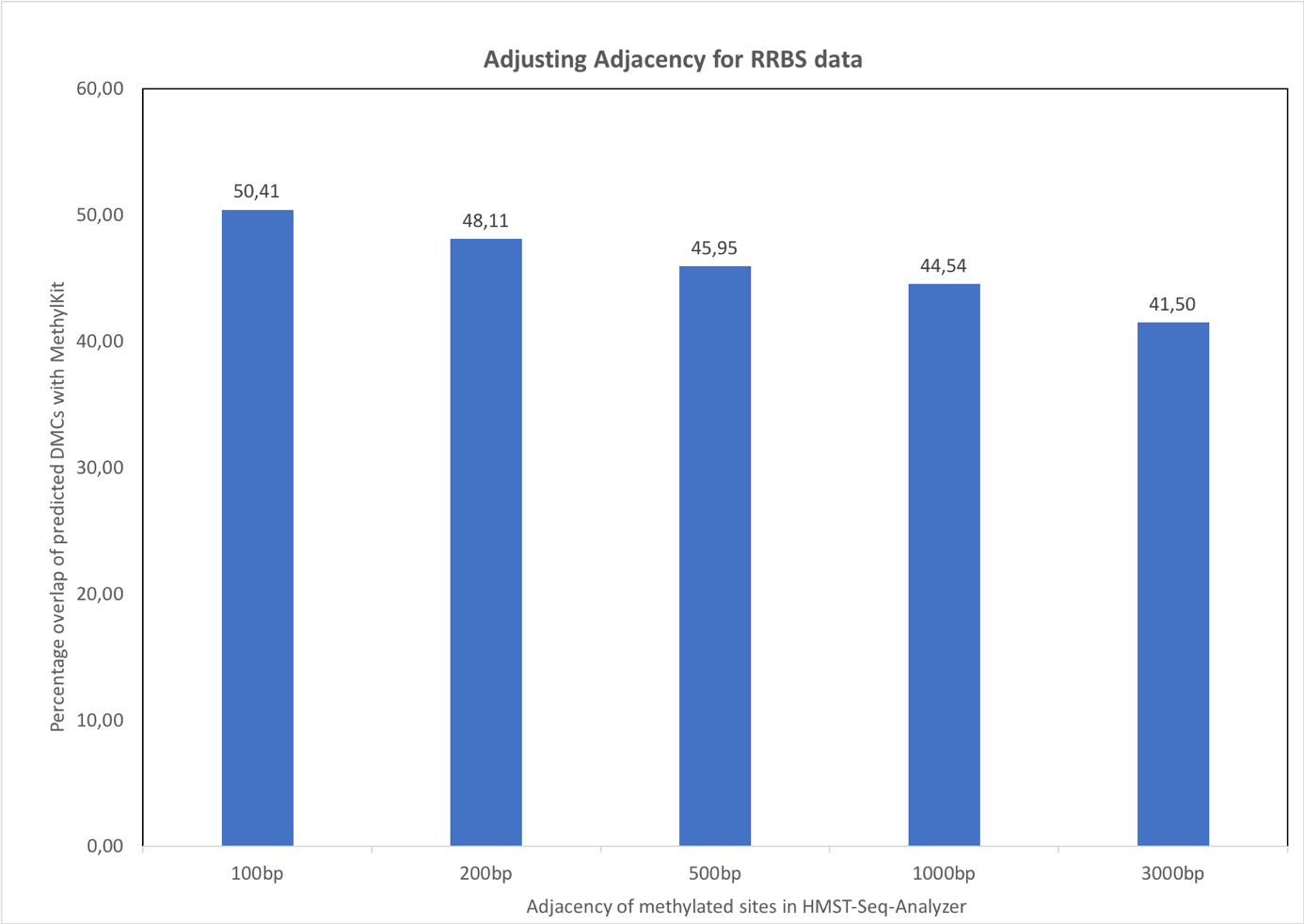


**SFigure 8: Frequency of the number of DMCs in promoter regions in MethylKit results for RRBS data (3938 genes).**

The number of differentially methylated CpG sites (DMCs) in each promoter region (+/-1Kb to TSS) is calculated based on RRBS data analysis using Methylkit. Here, ~61% of the promoters had only 1 DMC reported.


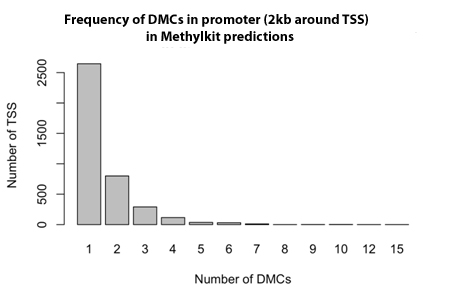


**SFigure 9: Frequency of the number of DMCs in promoter regions in Methylkit results for RRBS data (top 200).**

The number of differentially methylated CpG sites (DMCs) predicted by Methylkit (for RRBS data) in each promoter region is shown. Promoter regions were defined as 2kb around each TSS, and the top 200 promoters with the strongest DMCs were chosen on the basis of the P-value given by MethylKit. Here, ~70% of the promoters had only 1 DMC reported.

**
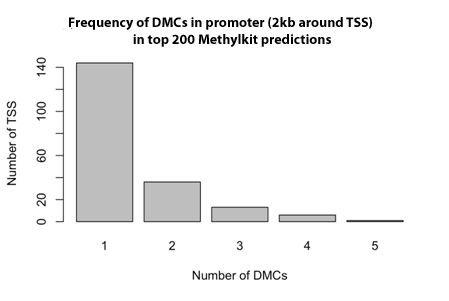
**

# **Supplementary Tables:**

# **STables 1 to 5 please refer to the attached Excel file.**

# **STable 1. Predicted 291 genes with DMCs in promoter regions (+/-1Kb to TSS) in HMST-Seq-Analyzer results for RRBS data.**

# **STable 2. Common 91 genes with DMCs in promoter regions (+/-1Kb to TSS) in both HMST-Seq-Analyzer and MethylKit results for RRBS data.**

# **STable 3. DAVID functional gene annotation results of 200 unique genes with DMCs in promoters from HMST-Seq-Analyzer results for RRBS data.**

# **STable 4. DAVID functional gene annotation results of top 200 unique genes with DMCs in promoters from MehtylKit results for RRBS data.**

# **STable 5. DAVID functional gene annotation results of top 500 unique genes with DMCs in promoters from MehtylKit results for RRBS data.**

# **STable 6. A comparison of HMST-Seq-Analyzer and three other packages in differentially methylated region analysis (chr17).**

|  | DMRs | Overlapping | Percentage  (%) | Length (bp) | Gene annotation (second) | DMR finding (second) | Total (second) |
| --- | --- | --- | --- | --- | --- | --- | --- |
| HMST-Seq-Analyzer | 2556 | 2556 | 100 | 10748 | 645 | 211 | 856 |
| BSmooth | 2253 | 1947 | 86 | 394 | NA | 134 | 134 |
| MethylSig | 72321 | 57983 | 80 | 200 | NA | 365 | 365 |
| MethylKit | 28201 | 22011 | 78 | 200 | NA | 172 | 172 |
| MethylSig | 103519 | 83610 | 81 | 25 | NA | 1092 | 1092 |
| MethylKit | 37272 | 29449 | 79 | 25 | NA | 233 | 233 |

HMST-Seq-Analyzer, BSmooth, MethylSig, and MethylKit were applied on the same WGBS data to identify differentially methylated regions (DMRs) between human H1 and IMR90 cells, respectively. Here, each cell line has replicated experiments and only chromosome 17 is used in the evaluation for all the programs. MethylKit and MethylSig are tested in two different lengths of window size (25bp and 200bp). In the table, DMRs represents the number of DMRs detected by the package. Overlapping and Percentage are the number of and the percentage of DMRs that are overlapping with the DMRs from HMST-Seq-Analyzer, respectively. Length is the median length of DMRs. DMR finding, Gene annotation and Total are wall time (seconds) used in each step, respectively.

# **STable 7. A comparison of HMST-Seq-Analyzer and three other packages in differentially methylated region analysis (chr1).**

|  | DMRs | Overlapping | Percentage  (%) | Length (bp) | Gene annotation (second) | DMR finding (second) | Total (second) |
| --- | --- | --- | --- | --- | --- | --- | --- |
| HMST-Seq-Analyzer | 7004 | 7004 | 100 | 11472 | 1322 | 446 | 1768 |
| BSmooth | 4498 | 3734 | 83 | 385 | NA | 426 | 426 |
| MethylSig | 214516 | 160039 | 75 | 200 | NA | 1062 | 1062 |
| MethylKit | 92556 | 67827 | 73 | 200 | NA | 430 | 430 |
| MethylSig | 288876 | 217746 | 75 | 25 | NA | 3712 | 3712 |
| MethylKit | 118405 | 88000 | 74 | 25 | NA | 602 | 602 |

HMST-Seq-Analyzer, BSmooth, MethylSig, and MethylKit were applied on the same WGBS data to identify differentially methylated regions (DMRs) between human H1 and IMR90 cells, respectively. Here, each cell line has replicated experiments and only chromosome 1 is used in the evaluation. In the table, DMRs represents the number of DMRs detected by the package. Overlapping and Percentage are the number of and the percentage of DMRs that are overlapping with the DMRs from HMST-Seq-Analyzer, respectively. Length is the median length of DMRs. DMR finding, Gene annotation and Total are wall time (seconds) used in each step, respectively.
